# Nutritional impact of adding a serving of mushrooms on usual intakes and nutrient adequacy using National Health and Nutrition Examination Survey 2011–2016 data

**DOI:** 10.1002/fsn3.2120

**Published:** 2021-01-12

**Authors:** Victor L. Fulgoni, Sanjiv Agarwal

**Affiliations:** ^1^ Nutrition Impact Battle Creek MI USA; ^2^ NutriScience LLC East Norriton PA USA

**Keywords:** crimini mushrooms, oyster mushrooms, portabella mushrooms, white mushrooms

## Abstract

To evaluate the nutritional impact of adding a serving of mushrooms on usual intakes and population adequacy of nutrients the National Health and Nutrition Examination Survey (NHANES) 2011–2016 dietary data for 9–18 years and 19+ years and a composite of commonly consumed raw mushrooms as well as oyster mushrooms (nutrient profiles from USDA data) were used for modeling. Usual intakes of nutrients and the percent population below the Estimated Average Requirement (EAR) or above the Adequate Intake (AI) were estimated before and after addition of mushrooms. Means with nonoverlapping 95th percentile confidence levels were used to assess meaningful differences. Addition of a serving (84 g) of mushrooms to the diet resulted in an increase in dietary fiber (5%–6%), copper (24%–32%), phosphorus (6%), potassium (12%–14%), selenium (13%–14%), zinc (5%–6%), riboflavin (13%–15%), niacin (13%–14%), and choline (5%–6%) in both adolescents and adults; and in iron (2.32%), thiamin (4.07%), folate (3.66%), and vitamin B_6_ (4.64%) in adults only, but had no impact on energy, carbohydrate, fat, or sodium. Addition of a serving of mushrooms also decreased the % below EAR for copper, phosphorus, and riboflavin for those 9–18 years and for copper, phosphorus, selenium, zinc, thiamine, riboflavin, niacin, folate, and vitamin B_6_ for those 19+ years and increased the % above AI for potassium for both age groups. Addition of oyster mushrooms additionally increased 12%–13% vitamin D, and 12%–15% choline in the NHANES 2011–2016 diets. Addition of mushrooms exposed to UV light to increase vitamin D levels to 5 µg/serving also almost doubled vitamin D intake (98%–104%) and decreased inadequacy. Addition of a serving of mushrooms would also add 2.2 mg ergothioneine and 3.5 mg glutathione to the diet. Addition of a mushroom serving to the diet would increase several micronutrients including shortfall nutrients, without having any impact on energy, sodium, or fat.

## Graphical abstract

Mushrooms are fungi and have long been part of the human diet. This modeling study evaluated the nutritional impact of adding mushrooms to current dietary intakes. Addition of mushrooms to NHANES 2011‐2016 increased several micronutrients including “underconsumed” and “shortfall nutrients” and had a minimal or no impact on calories, sodium, or fat. These results suggest that inclusion of mushrooms in the diets may be one effective approach to help Americans to reach dietary goals identified by Dietary Guidelines for Americans.

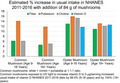

## 1 INTRODUCTION

Mushrooms are the fruiting bodies of filamentous fungi that grow above the ground, have long been a part of the human diet, and used as both foods and medicine (Alexopoulos et al., 1996; Davidson, 1999; Feeney et al., 2014). They are considered as vegetables and have been informally categorized among the “White Vegetables” from a culinary point of view (Weaver & Marr, 2013). Mushrooms are considered as part of “Other Vegetables” by USDA’s MyPlate (ChooseMyPlate.gov) and ½ cup of mushrooms counts as ½ cup equivalent in the Vegetable Group (Other Vegetables subgroup) (USDA, 2020). Mushrooms are low fat, low calorie foods and are an important source of nutrients and bioactive compounds. Mushrooms are generally rich in many B vitamins, selenium, copper, potassium, and fiber (Feeney, Dwyer, et al., 2014). They can also be an abundant source of vitamin D when exposed to UV light (Kalaras et al., 2012). Mushrooms contain a variety of phenolic antioxidants as secondary metabolites (Kalaras et al., 2017).

The National Health and Nutrition Examination Survey (NHANES), a continuous, very large, nationally representative cross sectional survey of noninstitutionalized U.S. population, is designed to monitor the dietary status and health of American children and adults (CDC, 2020). The survey examines a nationally representative sample of about 10,000 adults and children every two years and includes an in‐home survey and a mobile laboratory physical examination. The NHANES survey includes demographic, socioeconomic, dietary, and health‐related questions and assesses food consumption and dietary supplement use, and health‐related parameters of participants. Because NHANES surveys are conducted using the same standardized procedures, very large data sets can be obtained by combining multiple years of data. Data from these surveys are used to assess nutritional status and its association with health promotion and disease prevention and assist with formulation of national standards and public health policy (CDC, 2020).

Using NHANES 2001–2010 data, we earlier reported that mushroom intake was associated with higher intakes of several key nutrients and better diet quality; however, their intake was low at 2.3 g per day per capita or 20.6 g per day among consumers (O’Neil et al., 2013). In the present study, we modeled the nutritional impact of adding a serving of mushrooms using the dietary intake data from NHANES 2011–2016 surveys.

## 2 METHODS

### 2.1 Intake assessment

24‐hr dietary recall data from adolescents 9–18 years and adults 19+ years participating in NHANES for 2011–2012, 2013–2014, and 2015–2016 cycles were used. Data from pregnant or lactating females and those with unreliable or incomplete data determined by the USDA were excluded. A detailed description of the subject recruitment, survey design, and data collection procedures is available online (CDC, 2020), and all data obtained for this study are publicly available at: http://www.cdc.gov/nchs/nhanes/. NHANES protocol was approved by the National Center for Health Statistics (NCHS) Ethics Review Board and all participants or proxies provided a signed written informed consent. Energy and nutrient intake were obtained from the relevant USDA Nutrient Database for Standard Reference Releases in conjunction with the respective USDA Food and Nutrient Database for Dietary Studies (FNDDS) (Haytowitz et al., 2020; USDA/ARS, 2020a).

### 2.2 Mushroom composites

The following composites of raw mushrooms were created:

- Commonly consumed mushrooms: white + crimini + portabella at 1:1:1 ratio.
- “a” above exposed to UV light to increase vitamin D to 5 µg/serving.
- Specialty mushrooms: oyster mushrooms.

Nutrient profiles of mushrooms used were obtained from USDA Food Data Central database (USDA/ARS, 2020b) using the specific foods codes for each specific mushroom: white (mushroom, white, raw; FDC ID 169251), crimini (mushroom, brown, Italian or crimini, raw; FDC ID 168434), portabella (mushroom, portabella, raw; FDC ID 169255), and oyster (mushroom, oyster, raw; FDC ID 168580). Nutrient profiles for each mushroom composite were then computed for 84 g or 1/2 cup equivalent serving (Table 1).

**TABLE 1:** Nutrient Profiles of Mushrooms Composites (per 84 g serving) (USDA/ARS, 2020b)

	White + Crimini + Portabella Mushrooms at 1:1:1 ratio	Oyster Mushrooms
Energy (kcal)	18.5	27.7
Protein (g)	2.16	2.78
Carbohydrate (g)	3.20	5.12
Dietary Fiber (g)	0.81	1.93
Total Fat (g)	0.22	0.34
Saturated Fat (g)	0.02	0.05
Cholesterol (mg)	0.00	0.00
Calcium (mg)	6.72	2.52
Copper (mg)	0.31	0.20
Iron (mg)	0.34	1.12
Phosphorus (mg)	87.9	101
Potassium (mg)	316	353
Selenium (µg)	15.1	2.18
Sodium (mg)	5.6	15.1
Zinc (mg)	0.60	0.65
Vitamin A, RAE (µg)	0	1.68
Thiamin (mg)	0.07	0.105
Riboflavin (mg)	0.29	0.29
Niacin (mg)	3.33	4.16
Folate, DFE (µg)	19.6	31.9
Vitamin B_6_ (mg)	0.10	0.09
Vitamin B_12_ (µg)	0.05	0
Vitamin C (mg)	0.59	0
Vitamin D (µg)	0.14	0.61
Vitamin E (mg)	0.01	0
Choline (mg)	17.0	40.9

Note

Mushroom specific USDA Food Codes used were: white mushroom—FDC ID 169251; crimini mushroom—FDC ID 168434); portabella mushroom—FDC ID 169255; and oyster mushroom—FDC ID 168580. Vitamin D values for the mushrooms exposed to UV light were 5 µg.

### 2.3 Mushroom modeling

Dietary modeling was accomplished by adding nutrients from each mushroom composite to the NHANES 2011–2016 dietary intakes. Usual intakes of energy and nutrients were estimated using the National Cancer Institute Method V. 2.1 (Tooze et al., 2010); and the percentage of the population below the Estimated Average Requirement (EAR) or above Adequate Intake (AI) were estimated using the cut‐point method (except for iron) before and after addition of mushrooms. The probability method was used to determine population below EAR for iron because iron requirement distribution is highly skewed for in the case of menstruating women and young children (Institute of Medicine, 2000).

### 2.4 Statistics

All analyses were performed using SAS 9.4 (SAS Institute, Cary, NC, USA) software. The data were adjusted for the complex sampling design of NHANES, using day 1 dietary weights, strata, and primary sampling units. Means with nonoverlapping 95th percentile confidence levels were used to assess meaningful differences in usual intakes of energy and nutrients, and the percentage of the population below the EAR or above AI of nutrients before and after addition of mushrooms.

## 3 RESULTS

Addition of a serving (84 g) of commonly consumed mushrooms to the NHANES 2011–2016 diets resulted in an increase (nonoverlapping 95% CI) in dietary fiber (6.16%), copper (31.6%), phosphorus (6.46%), potassium (14.1%), selenium (13.6%), zinc (5.71%), riboflavin (14.6%), niacin (14.2%), and choline (6.11%), but had no impact on energy, carbohydrate, protein, fat, or sodium for adolescents 9–18 years; and an increase in dietary fiber (4.57%), protein (2.64%), copper (23.6%), iron (2.70%), phosphorus (6.34%), potassium (11.8%), selenium (12.9%), zinc (5.31%), thiamin (4.32%), riboflavin (13.4%), niacin (12.5%), folate (3.72%), vitamin B6 (4.61%), and choline (5.03%) for adults 19+ years. However, addition of commonly consumed mushrooms did not have any impact on energy, carbohydrate, fat, or sodium in NHANES 2011–2016 adult diets (Table 2).

**TABLE 2:** Estimates of mean of usual intakes of energy and nutrients in NHANES 2011–2016 without and with addition of 84 g of commonly consumed mushrooms (White + Crimini + Portabella mushrooms at 1:1:1) in adolescents 9–18 years (*n* = 4810) and adults 19+ years (*n* = 14990)

Nutrient	Adolescents 9–18 years		Adults 19+ years	
	Without mushroom addition	With mushroom addition	Without mushroom addition	With mushroom addition
Energy (kcal)	1997 (1959, 2034)	2013 (1976, 2050)	2,151 (2,133, 2,168)	2,169 (2,152, 2,186)
Carbohydrate (gm)	260 (255, 265)	263 (258, 268)	255 (252, 257)	258 (255, 260)
Dietary fiber (gm)	14.6 (14.3, 14.9)	15.5 (15.1, 15.8)	17.5 (17.1, 17.8)	18.3 (17.9, 18.6)
Protein (gm)	72.7 (71.1, 74.3)	74.7 (73.1, 76.4)	83.2 (82.3, 84.1)	85.4 (84.5, 86.3)
Total fat (gm)	75.8 (74.0, 77.5)	75.9 (74.1, 77.7)	83.3 (82.3, 84.3)	83.5 (82.6, 84.5)
Calcium (mg)	1,026 (997, 1,055)	1,032 (1,003, 1,061)	971 (958, 984)	978 (965, 991)
Copper (mg)	0.98 (0.96, 1.00)	1.29 (1.27, 1.31)	1.27 (1.25, 1.29)	1.57 (1.55, 1.59)
Iron (mg)	14.7 (14.3, 15.1)	15.0 (14.7, 15.4)	14.8 (14.7, 15.0)	15.2 (15.0, 15.3)
Phosphorus (mg)	1,316 (1,286, 1,346)	1,401 (1,372, 1,431)	1,403 (1,388, 1,417)	1,492 (1,477, 1508)
Potassium (mg)	2,214 (2,168, 2,260)	2,526 (2,479, 2,572)	2,694 (2,661, 2,728)	3,012 (2,979, 3,045)
Selenium (µg)	103 (100, 105)	117 (115, 120)	116 (115, 118)	131 (130, 132)
Sodium (mg)	3,262 (3,186, 3,337)	3,268 (3,194, 3,341)	3,559 (3,525, 3,594)	3,566 (3,531, 3,600)
Zinc (mg)	10.5 (10.2, 10.7)	11.1 (10.8, 11.3)	11.3 (11.2, 11.5)	11.9 (11.8, 12.1)
Vitamin A, RAE (µg)	581 (560, 602)	581 (560, 602)	643 (625, 662)	643 (624, 662)
Thiamin (mg)	1.61 (1.57, 1.65)	1.67 (1.63, 1.72)	1.62 (1.60, 1.64)	1.69 (1.67, 1.70)
Riboflavin (mg)	1.98 (1.94, 2.03)	2.27 (2.22, 2.32)	2.17 (2.15, 2.20)	2.46 (2.43, 2.48)
Niacin (mg)	23.2 (22.6, 23.9)	26.5 (25.9, 27.2)	26.3 (26.0, 26.7)	29.6 (29.3, 30.0)
Folate, DFE (µg)	530 (514, 547)	550 (533, 567)	538 (530, 546)	558 (550, 566)
Vitamin B6 (mg)	1.82 (1.76, 1.88)	1.92 (1.86, 1.98)	2.17 (2.13, 2.20)	2.27 (2.23, 2.30)
Vitamin B12 (µg)	4.85 (4.69, 5.01)	4.89 (4.74, 5.05)	5.09 (4.98, 5.20)	5.15 (5.04, 5.26)
Vitamin C (mg)	68.2 (64.8, 71.5)	68.8 (65.4, 72.2)	80.9 (78.1, 83.8)	81.7 (78.8, 84.5)
Vitamin D (µg)	5.10 (4.88, 5.32)	5.23 (5.02, 5.45)	4.68 (4.56, 4.81)	4.82 (4.70, 4.94)
Vitamin E (mg)	7.38 (7.16, 7.60)	7.39 (7.17, 7.61)	9.18 (8.97, 9.38)	9.19 (8.98, 9.40)
Total choline (mg)	262 (255, 268)	278 (272, 285)	338 (335, 342)	355 (3.51, 359)

Note

Data presented as Means (95% Confidence Interval). Nutrient data on mushrooms were used from Table 1. One serving (84 g) of mushrooms was added to all diets of those 9–18 years and 19 + years. Usual intakes of energy and nutrients were estimated using the National Cancer Institute (NCI) Method V. 2.1 (Tooze et al., 2010). Nonoverlapping 95% confidence intervals were used to assess difference between without and with addition of mushrooms.

Addition of commonly consumed mushrooms exposed to UV light to increase vitamin D to 5 µg/serving in NHANES 2011–2016 diets also increased (nonoverlapping 95% CI) vitamin D by 98.0% from 5.10 (95% CI: 4.89, 5.32) to 10.1 (95% CI: 9.92, 10.3) µg for those 9–18 years and by 104% from 4.68 (95% CI: 4.55, 4.81) to 9.53 (95% CI: 9.41, 9.64) µg for those 19+ years. Similar to commonly consumed mushrooms, addition of oyster mushrooms to the diets also increased (nonoverlapping 95% CI) fiber, copper, iron, phosphorus, potassium, zinc, thiamine, riboflavin, niacin, folate, and vitamin B_6_, but did not affect energy, fat, or sodium. However, addition of oyster mushrooms additionally increased (nonoverlapping 95% CI) vitamin D (11.7% for those 9–18 years; and 12.6% for those 19+ years) and choline (15.3% for those 9–18; and 12.1% for those 19+ years) in the NHANES 2011–2016 diets (Table 3).

**TABLE 3:** Estimates of mean usual intakes of energy and nutrients in NHANES 2011–2016 without and with addition of 84 g of oyster mushrooms in adolescents age 9–18 years (*n* = 4,810) and adults age 19 + years (*n* = 14,990)

Nutrient	Adolescents 9–18 years		Adults 19 + years	
	Without mushroom addition	With mushroom addition	Without mushroom addition	With mushroom addition
Energy (kcal)	1998 (1961, 2034)	2023 (1986, 2059)	2,150 (2,133, 2,167)	2,177 (2,161, 2,193)
Carbohydrate (gm)	260 (255, 265)	265 (260, 270)	255 (252, 257)	260 (257, 262)
Dietary fiber (gm)	14.6 (14.3, 14.9)	16.5 (16.2, 16.9)	17.5 (17.1, 17.8)	19.4 (19.0, 19.7)
Protein (gm)	72.7 (71.0, 74.3)	75.4 (73.7, 77.0)	83.2 (82.3, 84.1)	86.0 (85.1, 86.9)
Total fat (gm)	75.8 (74.0, 77.5)	76.1 (74.3, 77.9)	83.3 (82.3, 84.3)	83.6 (82.7, 84.6)
Calcium (mg)	1,026 (997, 1,055)	1,029 (1,000, 1,057)	971 (958, 984)	973 (960, 986)
Copper (mg)	0.98 (0.96, 1.00)	1.19 (1.17, 1.21)	1.27 (1.25, 1.29)	1.47 (1.45, 1.49)
Iron (mg)	14.7 (14.3, 15.1)	15.8 (15.4, 16.2)	14.8 (14.7, 15.0)	15.9 (15.8, 16.1)
Phosphorus (mg)	1,315 (1,285, 1,345)	1,414 (1,384, 1,444)	1,403 (1,389, 1,417)	1504 (1,489, 1518)
Potassium (mg)	2,212 (2,166, 2,258)	2,564 (2,517, 2,610)	2,694 (2,661, 2,728)	3,048 (3,015, 3,081)
Selenium (µg)	103 (100, 105)	105 (102, 107)	116 (115, 118)	118 (117, 120)
Sodium (mg)	3,264 (3,190, 3,338)	3,279 (3,204, 3,353)	3,561 (3,526, 3,595)	3,577 (3,542, 3,611)
Zinc (mg)	10.5 (10.2, 10.8)	11.1 (10.9, 11.4)	11.4 (11.2, 11.5)	12.0 (11.9, 12.1)
Vitamin A, RAE (µg)	581 (560, 602)	583 (562, 605)	643 (625, 662)	645 (626, 664)
Thiamin (mg)	1.61 (1.57, 1.65)	1.71 (1.67, 1.76)	1.62 (1.60, 1.64)	1.72 (1.71, 1.74)
Riboflavin (mg)	1.99 (1.94, 2.04)	2.27 (2.22, 2.33)	2.17 (2.15, 2.20)	2.46 (2.44, 2.49)
Niacin (mg)	23.2 (22.6, 23.9)	27.3 (26.7, 28.0)	26.3 (25.9, 26.7)	30.4 (30.1, 30.8)
Folate, DFE (µg)	530 (513, 546)	562 (545, 579)	538 (530, 546)	570 (562, 579)
Vitamin B6 (mg)	1.82 (1.76, 1.88)	1.91 (1.85, 1.97)	2.17 (2.13, 2.21)	2.26 (2.22, 2.30)
Vitamin B12 (µg)	4.86 (4.70, 5.02)	4.84 (4.67, 5.00)	5.09 (4.98, 5.21)	5.09 (4.98, 5.20)
Vitamin C (mg)	68.2 (64.8, 71.7)	68.3 (64.8, 71.7)	80.9 (78.1, 83.7)	81.0 (78.2, 83.8)
Vitamin D (µg)	5.11 (4.89, 5.32)	5.71 (5.50, 5.93)	4.69 (4.56, 4.81)	5.28 (5.15, 5.40)
Vitamin E (mg)	7.38 (7.16, 7.60)	7.38 (7.15, 7.60)	9.18 (8.98, 9.38)	9.18 (8.98, 9.38)
Total choline (mg)	262 (255, 268)	302 (295, 309)	338 (334, 342)	379 (375, 382)

Note

Data presented as Means (95% Confidence Interval). Nutrient data on mushrooms were used from Table 1. One serving (84 g) of mushrooms was added to all diets of those 9–18 years and 19+ years. Usual intakes of energy and nutrients were estimated using the National Cancer Institute (NCI) Method V. 2.1 (Tooze et al., 2010). Nonoverlapping 95% confidence intervals (indicated by yellow highlighting) were used to assess difference between without and with addition of mushrooms.

Addition of a serving of commonly consumed mushrooms to NHANES 2011–2016 diets decreased (nonoverlapping 95% CI) the population inadequacy (% below EAR) for copper, phosphorus, and riboflavin for age 9–18 years, and for copper, phosphorus, selenium, zinc, thiamine, riboflavin, niacin, folate, and vitamin B_6_, for age 19 + years (Table 4). Population % above AI for potassium also increased (nonoverlapping 95% CI) for both age groups with addition of commonly consumed mushrooms to NHANES 2011–2016 diets. A serving of UV light exposed commonly consumed mushrooms also decreased population inadequacy (% below EAR) for vitamin D from 95.3% (95% CI: 94.1%, 96.4%) to 52.8% (95% CI: 49.4%, 56.2%) for age group 9–18 years and from 94.9% (95% CI: 94.2%, 95.7%) to 63.6% (95% CI: 61.6%, 65.6%) for age group 19+ years.

**TABLE 4:** Estimates of population inadequacy/adequacy (% below EAR or above AI) of nutrients in NHANES 2011–2016 diet before and after addition of 84 g of commonly consumed mushrooms (White + Crimini + Portabella mushrooms at 1:1:1) in adolescents age 9–18 years (*n* = 4,810) and adults age 19+ years (*n* = 14,990)

Nutrients	Adolescents 9–18 years		Adults 19+ years	
	Without mushroom addition	With mushroom addition	Without mushroom addition	With mushroom addition
Nutrients with Estimated Average Requirement (EAR)				
Calcium	62.5 (59.2, 65.8)	62.1 (58.7, 65.5)	43.3 (41.7, 44.9)	42.6 (41.0, 44.2)
Copper	7.68 (5.76, 9.60)	0.06 (0.00, 0.14)	7.03 (6.25, 7.81)	0.20 (0.13, 0.26)
Iron	3.63 (2.65, 4.62)	3.05 (2.15, 3.95)	5.34 (4.85, 5.84)	4.68 (4.24, 5.12)
Phosphorus	23.9 (20.5, 27.3)	15.8 (12.8, 18.8)	0.71 (0.52, 0.90)	0.20 (0.13, 0.27)
Selenium	0.11 (0.00, 0.22)	0.00 (0.00, 0.00)	0.51 (0.33, 0.68)	0.02 (0.00, 0.03)
Zinc	15.8 (12.4, 19.3)	10.0 (7.1, 12.9)	16.9 (15.2, 18.7)	11.6 (10.1, 13.2)
Vitamin A, RAE	38.9 (34.7, 43.0)	38.8 (34.6, 43.0)	44.5 (42.2, 46.8)	44.6 (42.3, 46.9)
Thiamin	2.11 (0.89, 3.32)	1.14 (0.37, 1.92)	7.02 (5.98, 8.07)	4.60 (3.75, 5.45)
Riboflavin	1.58 (0.61, 2.55)	0.10 (0.00, 0.22)	2.97 (2.52, 3.43)	0.36 (0.26, 0.46)
Niacin	0.43 (0.01, 0.85)	0.01 (0.00, 0.02)	1.44 (1.13, 1.76)	0.08 (0.04, 0.12)
Folate, DFE	6.58 (4.06, 9.10)	4.61 (2.50, 6.71)	12.8 (11.4, 14.3)	9.84 (8.57, 11.1)
Vitamin B6	3.27 (1.38, 5.16)	1.46 (0.35, 2.56)	10.7 (9.62, 11.8)	7.23 (6.32, 8.14)
Vitamin B12	1.66 (0.72, 2.60)	1.50 (0.73, 2.28)	4.75 (3.91, 5.60)	4.22 (3.41, 5.03)
Vitamin C	35.3 (31.3, 39.3)	34.8 (30.8, 38.8)	47.5 (45.3, 49.7)	47.2 (45.0, 49.4)
Vitamin D	95.2 (94.1, 96.4)	95.0 (93.7, 96.2)	94.9 (94.2, 95.7)	94.5 (93.7, 95.3)
Vitamin E	87.0 (84.0, 89.9)	87.0 (84.0, 90.0)	79.9 (78.0, 81.8)	79.8 (77.8, 81.7)
Nutrients with Adequate Intake (AI)				
Dietary fiber	0.37 (0.19, 0.56)	0.63 (0.36, 0.90)	8.62 (7.52, 9.72)	10.1 (8.87, 11.3)
Potassium	27.7 (25.0, 30.4)	47.2 (44.1, 50.3)	32.2 (30.5, 34.0)	47.8 (46.0, 49.7)
Sodium	99.9 (99.8, 100)	99.1 (99.8, 100)	99.3 (99.1, 99.4)	99.3 (99.1, 99.4)
Total choline	4.52 (3.31, 5.74)	6.36 (4.89, 7.83)	8.04 (6.87, 9.22)	10.2 (8.96, 11.4)

Note

Data presented as Means (95% Confidence Interval). Nutrient data on mushrooms from Table 1 were used. One serving (84 g) of mushrooms was added to all diets of those 9–18 years and 19+ years. Population adequacy/inadequacy were estimated using the cut‐point method (except for iron where the probability method was used) before and after addition of mushrooms. Nonoverlapping 95% confidence intervals were used to assess difference between without and with addition of mushrooms.

## 4 DISCUSSION

Results of this NHANES 2011–2016 modeling study indicate that addition of mushrooms to the diet increased the intake and decreased population inadequacy of several key micronutrients without affecting intake of calories, fat or sodium and thus have beneficial effects on the diets of children (9–18 years) and adults (19+ years).

Inadequate intake of micronutrients has been consistently associated with adverse health effects such as neural tube defects, poor bone health (osteoporosis), impaired immune function, and impaired cognitive function, as well as chronic diseases, such as certain cancers, age‐related eye diseases, hypertension, and possibly coronary heart disease and stroke (Ames, 2006; CDC, 2012; Fairfield & Fletcher, 2002). According to the CDC’s Second National Report on Biochemical Indicators of Diet and Nutrition in the U.S. Population, nearly 10% of the U.S. population had nutritional deficiencies (based on nutrient status markers), and could be as high as nearly one third for certain subpopulation groups (CDC, 2012). 2015–2020 Dietary Guidelines for Americans (DGA) has identified calcium, potassium, iron (adolescent and adult females), magnesium, dietary fiber, choline, and vitamins A, D, E, and C as "underconsumed nutrients" since their intakes for many individuals are below the recommendations and vitamin D, calcium, iron, potassium, and fiber as "nutrients of public health concern” (USDHHS/USDA, 2020). The Scientific Report of the 2020 Dietary Guidelines Advisory Committee (2020 DGAC Report) reaffirmed vitamin D, calcium, dietary fiber, and potassium are underconsumed, and sodium, saturated fat, and added sugars are overconsumed; and are of public health concern for all Americans (Dietary Guidelines Advisory Committee, 2020). Mushrooms are rich sources of several micronutrients (Feeney, Dwyer, et al., 2014) and adults who consumed mushrooms had higher diet quality with higher intakes of fiber, vitamin E, riboflavin, niacin, folic acid vitamin C, choline, copper, potassium, and selenium, than those who did not consume mushroom in our earlier analysis of NHANES 2001–2010 (O’Neil et al., 2013). In the present modeling analysis of NHANES 2011–2016 diets, addition of a serving of mushrooms increased the amounts of dietary fiber, copper, iron, phosphorus, potassium, selenium, zinc, thiamine, riboflavin, niacin, folate, vitamin B6, and choline and increased population adequacy for several of these nutrients. Interestingly, calories, fat, or sodium were unaffected by addition of a serving of mushroom in this modeling analysis.

Mushroom are also rich sources of ergosterol, which is a precursor of vitamin D (Jasinghe & Perera, 2005; Mattila et al., 2002; Phillips et al., 2011). With exposure to UV light, mushroom ergosterol is dose dependently converted to ergocalciferol and increase mushroom vitamin D_2_ contents (Cardwell et al., 2018; Jasinghe & Perera, 2006; Jasinghee et al., 2007; Roberts et al., 2014, 2008). Exposure to natural sunlight for 15 min was also found to increase vitamin D_2_ contents of mushrooms by 150 to >600 IU/70 mg or 25 to >100% EAR in a preliminary study (Phillips & Rasor, 2013). Exposure to UV light in controlled conditions is more commonly used to provide desired amounts on vitamin D in mushrooms (Cardwell et al., 2018; Jaisinghe et al., 2007; Jasinghe & Perera, 2005, 2006; Phillips et al., 2011; Roberts et al., 2014, 2008. Vitamin D_2_ from UV light exposed mushrooms has been shown to be bioavailable and increase serum vitamin D status in human studies (Outila et al., 1999; Urbain et al., 2011). In our modeling analysis, addition of a serving of UV light exposed mushrooms to NHANES 2011–2016 diets resulted in about 100% increase in vitamin D in the diets and decreased population inadequacy from 95% to 53% in those 9–18 years and to 64% in those 19+ years. Some common edible varieties of mushrooms are also rich natural sources of vitamin D (Cardwell et al., 2018; Jasinghe & Perera, 2005; Phillips et al., 2011), and interestingly, addition oyster mushrooms (specialty mushrooms) increased vitamin D in the diet by 12%–13%.

Mushrooms are rich sources of critical bioactive phytonutrients in addition to important vitamins and minerals. They are also one of the best dietary sources of sulfur containing antioxidant amino acid ergothioneine and tripeptide glutathione (Dubost et al., 2006; Halliwell et al., 2018; Kalaras et al., 2017; Pizzorno, 2014, 2008). Ergothioneine and glutathione contents in mushrooms very considerably depending upon the mushroom varieties, and oyster mushrooms contains significantly more amounts of these sulfur containing antioxidants than commonly consumed mushrooms: white button, crimini, or portabella mushrooms (Dubost et al., 2006; Kalaras et al., 2017). USDA nutrient database and USDA Food Data Central do not include analytical data on these novel antioxidants (Haytowitz et al., 2020; USDA/ARS, 2020b). Addition of a serving of commonly consumed mushrooms and oyster mushrooms would be expected to add 2.24 and 24.0 mg ergothioneine, respectively, and 3.53 and 12.3 mg glutathione, respectively, to the NHANES 2011–2016 diets based on published literature values (Dubost et al., 2006; Kalaras et al., 2017).

Strengths of this study include the use of a large, nationally representative database to assess food and nutrient intakes, and the same‐person modeling used in this study complements past studies based on consumers and nonconsumers of a specific food. There were a number of limitations in this study. The intake data from NHANES are self‐reported which rely on memory and are therefore subject to reporting bias (Subar et al., 2015). The results presented are based on modeling to evaluate the maximum effect of adding mushrooms and may not reflect actual individual dietary behavior; however, such modeling offers a technique to test potential nutritional impact of dietary guidance.

In conclusion, the results of this modeling study provide insight into the nutritional benefits of adding mushrooms to the current dietary intakes. Addition of mushrooms to NHANES 2011–2016 diets increased several micronutrients including “underconsumed” and “shortfall nutrients” and had a minimal or no impact on overall calories, sodium, or fat. These results suggest that inclusion of mushrooms in the diets may be one effective approach to help Americans to reach dietary goals identified by DGA (USDHHS/USDA, 2020).

## ETHICAL APPROVAL

Victor L Fulgoni as Senior Vice President of Nutrition Impact, LLC performs consulting and database analyses for various food and beverage companies and related entities, and Sanjiv Agarwal as Principal of NutriScience LLC performs nutrition science consulting for various food and beverage companies and related entities. The study was supported by the Mushroom Council. The data collected for this study conforms to all US regulations and guidelines regarding human subjects and all NHANES protocols were approved by the National Center for Health Statistics (NCHS) Ethics Review Board. All subjects or proxies provided written informed consent to participate in the study.

## Data Availability

The US data used in the article are publicly available at the NHANES website: https://www.cdc.gov/nchs/nhanes/index.htm
